# Dependence of Thyroid Sonographic Markers of Malignancy and Its Influence on the Diagnostic Value of Sonographic Findings

**DOI:** 10.1155/2015/693404

**Published:** 2015-01-22

**Authors:** Kosma Woliński, Adam Stangierski, Ewelina Szczepanek-Parulska, Edyta Gurgul, Marek Ruchała

**Affiliations:** Department of Endocrinology, Metabolism and Internal Medicine, Poznan University of Medical Sciences, 49 Przybyszewskiego Street, 60-355 Poznan, Poland

## Abstract

*Introduction*. Thyroid nodules constitute frequent medical condition. Ultrasonographic (US) examination remains the basis in the diagnostics of nodular goiter and selection of the suspected ones requiring fine-needle aspiration biopsy (FNAB). The aim of this study was to evaluate if the features so far considered to be US malignancy markers are dependent or independent variables and to check if these data are clinically relevant. *Materials and Methods*. Patients with diagnosed thyroid nodular goiter admitted for thyroidectomy, irrespectively of the indications for surgery, were involved. The following parameters were assessed: echogenicity, the presence of calcifications, presence of halo, shape, margins, structure (solid, partially or pure cystic), and elasticity of the nodules (assessed quantitatively). *Results*. 122 consecutive patients with 393 thyroid nodules were included. There were significant associations between halo absence and irregular borders, micro- and macrocalcifications, taller-than-wide feature and macrocalcifications, irregular margins and macrocalcifications, and also decreased elasticity of nodules and several attributes (partially cystic character, micro- and macrocalcifications). *Conclusions*. Not only diagnostic value of particular sonographic features but also data about cooccurrence and associations between them are clinically relevant. Although most of these features turned out to be independent, omitting significant association can lead to incorrect assessment of the risk of malignancy.

## 1. Introduction

Thyroid nodular disease constitutes frequent medical condition. According to numerous studies it affects 10 to 67% of general adult population and is even more common among several groups of subjects such as elderly, women, or acromegalic patients [1–6]. Ultrasonographic (US) examination remains the gold standard in the preliminary assessment of thyroid lesions and selection of those requiring fine-needle aspiration biopsy (FNAB) [7–9].

Recently sonoelastography, a technique of tissue stiffness assessment, was widely described as a method significantly improving classical ultrasonography. According to numerous studies, malignant lesions are significantly stiffer than benign ones [10–16].

There were numerous studies on the topic of US markers of malignancy and panels of combined markers in the differentiation between benign and malignant thyroid lesions [8]. In most of them these features were implicitly considered to be independent. However to our knowledge the issue of dependence of sonographic markers of malignancy has not been comprehensively explored. This issue is of vital importance in endocrine practice as it is crucial for estimation of malignancy risk on the basis of US characteristics of the lesions. Theoretically presence of two independent markers of malignancy multiplies the relative risk of malignancy; in case of two strongly associated features the risk should be near to this of the stronger marker.

The aim of this study was to evaluate if the features so far considered to be US malignancy markers are dependent or independent variables and to check if such data is important in constructing panels of sonographic markers of malignancy.

## 2. Materials and Methods

### 2.1. Ethics Statement

The study was approved by the local Bioethical Committee of Poznan University of Medical Sciences. All participants provided informed written consent to participate in it.

### 2.2. Patients

Patients with diagnosed thyroid nodular goiter admitted for thyroidectomy between June and December 2010 irrespectively of the indications for surgery were involved. One hundred and twenty-two patients met the inclusion criteria and were enrolled in the study. The study was approved by local ethical committee.

### 2.3. Ultrasonography and Elastography

Conventional US and Shear-Wave Elastography (SWE) were performed with AIXPLORER system by Supersonic Imagine. The following parameters of particular lesions were assessed: echogenicity (hypo-, hyper-, and isoechogenic), the presence of calcifications (micro or macro), presence of halo (hypoechogenic rim), shape (oval, round, or “taller than wide”), margins (well defined or diffused), composition (solid, predominantly solid, predominantly cystic, or cystic), and elasticity of the nodules (assessed quantitatively; the mean stiffness of each nodule, so called Q-box mean, expressed in kPa was used in further calculations). The final diagnosis of the character of thyroid nodules (benign or malignant) was based on a histological examination performed as a routine medical procedure after surgery. The detailed characteristics of the group are available in the previously published paper [10].

### 2.4. Statistical Analysis

All calculations were performed using Statistica 10 (StatSoft). The *P* level of less than 0.05 was considered statistically significant. The significance of association between qualitative sonographic markers was evaluated using Fisher's exact test. Significance of difference between mean elasticity value between groups of nodules was calculated by *t*-test for independent samples.

### 2.5. Design

In the first step we have calculated relative risks (RRs) of malignancy for lesions possessing particular US features. This parameter was chosen as primary for further calculations, as theoretically presence of two independent markers of malignancy should multiply the RR of malignancy.

In the second step we have assessed the cooccurrence of particular markers of malignancy. In this stage only benign lesions were selected in order to avoid associations caused entirely by the fact that features considered as markers of malignancy frequently coexist in malignant lesions.

In the third step we have constructed all possible panels composed of two markers, in order to assess directly the influence of dependence of markers on the diagnostic values of particular panels.

In the fourth step of calculations we have evaluated diagnostic value of panels composed of three or more markers of malignancy.

## 3. Results

One hundred and twenty-two consecutive patients (103 men, 19 women) with 393 thyroid nodules were included. Mean age was 51.0 with standard deviation equal to 13.6 years. Twenty-two nodules in 22 patients were diagnosed as malignant by histopathology (18 papillary, two follicular, one medullary, and one anaplastic thyroid cancer).

Relative risks of malignancy for lesions possessing particular recorded features are presented in Table 1. For elastography the threshold of mean elasticity ≥38 kPa was selected as the highest RR was achieved for this cut-off point. Results of calculations on the dependence of sonographic features are shown in Table 2. Most of them turned out to be independent. However there were significant associations between halo absence and irregular borders, micro- and macrocalcifications, taller-than-wide feature and macrocalcifications, irregular margins and macrocalcifications, decreased elasticity of nodules, and several attributes (partially cystic character, micro- and macrocalcifications).

In the third step to evaluate the clinical significance of the dependence of sonographic features we constructed all possible panels composed of two US features (shown in Table 3).

In the fourth step we have assessed panels composed of three and four markers of malignancy. Selected panels are shown in Table 4.

## 4. Discussion

According to our results although most US markers of malignancy turned out to be independent some of them are significantly associated. One of the noted associations was common coexistence of halo absence and irregular margins. The explanation of this phenomenon could be the fact that the presence of halo, by definition, is associated with regularity of margins. Other features which turned out to be significantly associated were calcifications and decreased elasticity. As calcifications are rigid masses this observation seems to be quite easy to explain. Thyroid lesions with calcifications, especially coarse ones, were previously described as not suitable group for elastographic examination due to increased stiffness and false-positive results of the assessment of risk of malignancy [17, 18]. In our study, also a significant association between lesion elasticity and character of the lesion was found; lesions containing cystic components were less elastic than solid ones. In the context of this finding the use of elastography in case of partially cystic nodules seems to be controversial. Similar phenomenon was described by Bhatia et al. [19]. The explanation of this observation remains not fully understood. One of the previously suggested potential causes is the pressure exerted by the liquid of cystic compartment; the second possible explanation was that this result was some kind of artifact [19]. Bhatia et al. hypothesized that this effect could result from uneven stress distribution or by the fact that as elastograms are spatial maps of relative strain within the elastography window low signal within fluid in cystic nodules may have resulted in falsely increased signal in other tissues including the solid portion of cystic nodules.

The background of another observed association, between micro- and macrocalcifications, could be the fact that the threshold of 2 mm size between micro- and macrocalcifications is in fact arbitral. In our study in case of lesions with macrocalcifications microcalcifications were also identified in half of cases.

Association of macrocalcifications and irregular margins or taller than wide feature is difficult to interpret and requires further investigation. One possible explanation could be the fact that calcifications of relatively large size could influence the shape of the lesion. Moreover the calcification localized near the borders of the lesion by generating acoustic shadows might cause the impression of blurred margins.

Despite the fact that the background of association of some features remains not completely clear, it influences strongly the diagnostic value of the panels of markers of malignancy and should be taken into account in the assessment of the character of thyroid lesions. This effect can be clearly seen on the example of sets of two markers. Relative risks of malignancy for panels consisting of independent features are near the RR of one marker multiplied by RR of the second one (e.g., decreased elasticity, 3.07, irregular margins, 2.81, and both features together, 7.23). In consequence presence of two even moderately strong markers increases the risk of malignancy strongly. Conversely in case of two dependent markers of malignancy sensitivity should be near to this of the less sensitive one, specificity near to this of the more specific feature. In most cases the RR will be near to this of the stronger of two markers. For example RR for coexisting halo absence and irregular margins is 2.84, whereas for irregular margins alone it is 2.81. Similarly RR for increased stiffness and macrocalcifications is equal to 3.75 and for decreased elasticity alone it is 3.07. In the context of our results considering nodules with two strongly dependent sonographic features of malignancy as highly suspiciousmight lead to overestimation of the risk of malignancy, for example, inappropriate selection of lesions for biopsy in case of multinodular goiter.

Also analysis of panels composed of three or four markers brought interesting results. Most valuable panels of markers in concordance with previous predictions were composed of features with highest RR especially decreased elasticity which turned out to be the strongest marker of malignancy (Table 4). In cases of independent markers of malignancy lesions possessing four disquieting features were at higher risk of malignancy than those possessing only three of these features. In case of panels containing dependent features the results were different. Somehow surprisingly in most cases lesions possessing three suspected features were at similar or even higher risk of malignancy than nodules with same three features and additional fourth one, dependent from any other from the panel (Table 4). For example RR for lesions with decreased elasticity, hypoechogenicity, and irregular margins was equal to 12.6, whereas ones with all above features and macrocalcifications had RR equal to 4.2. Background of this finding is not entirely clear. Although our group of patients can be considered large number of thyroid cancers is too low to analyze precisely properties of panels composed of three or four features, which present low sensitivity, and in case of most panels only few of thyroid cancers in our group possessed them. This intriguing fact should be interpreted partially from statistical and partially from pathophysiological point of view. Taking into account the first approach it is important to consider the properties of dependent variables. If one feature was completely dependent on another, what means that every nodule with feature A possessed feature B (but not conversely), panel composed of these two markers would have sensitivity of the less sensitive one and specificity of the more specific one. If feature B was less sensitive more specific (occurs less often in benign lesions) diagnostic value of the panel would be equal to this of the feature B; inclusion of feature A has no effect. In our study similar situation occurred in case of halo absence and irregular margins. Interpretation of our findings from pathophysiological point of view is more sophisticated. Theoretically two independent features coexist randomly. Dependent features have probably some common background. For example if we take into account nodules with macrocalcifications and decreased elasticity we can expect that there will be mainly lesions with large, coarse, or numerous calcifications increasing stiffness of the nodule. In consequence taking into account these two features together is in fact not adding two markers of malignancy but selecting some particular subgroup of macrocalcifications.

In conclusion, most of analyzed US features of malignancy turned out to be independent; however some of them were significantly associated (halo absence and irregular borders; macrocalcifications and several attributes, microcalcifications, taller-than-wide feature, and irregular margins; decreased elasticity of nodules and partially cystic character, micro- and macrocalcifications). Somehow surprisingly in most cases lesions possessing three suspected features were at similar or even higher risk of malignancy than nodules with same three features and additional fourth one dependent from any other from the panel. In the view of our results not only diagnostic value of particular sonographic features but also data about cooccurrence and associations between them are clinically relevant and should be taken into consideration when estimating malignancy risk.

## Figures and Tables

**Table 1:** Relative risks of malignancy for lesions possessing particular markers of malignancy assessed in our study.

Feature	Relative risk
Hypoechogenicity	1.48 [1.31–1.67]
Microcalcifications	2.17 [1.26–3.74]
Macrocalcifications	2.81 [1.21–6.53]
Taller than wide	3.75 [1.73–8.12]
Halo absence	1.11 [1.01–1.23]
Solid character	1.49 [1.24–1.80]
Irregular margins	2.81 [2.06–3.83]
Mean elasticity ≥38 kPa	3.07 [2.50–3.76]

**Table 2:** Statistical significance of associations between particular features. Values given—*P* value; percentage of lesions with the feature in line possessing the feature from column; percentage of lesions with feature from line without the feature from column. For elasticity, *P* value was given for the difference between two means.

	Taller than wide	Hypoechogenicity	Halo absence	Irregular margins	Elasticity	Solid character	Microcalcifications	Macrocalcifications
Taller than wide	**X**	*P* = 0.42. 58.6 versus 66.6%	*P* = 0.40. 92.7 versus 85.7%	*P* = 0.51. 31.0 versus 35.5%	*P* = 0.89	*P* = 0.43. 67.9 versus 58.5%	*P* = 0.21. 28.6 versus 7.4%	*P* = 0.01. 20.7 versus 6.1%

Hypoechogenicity	*P* = 0.42. 6.7 versus 9.2%	**X**	*P* = 0.54. 84.7 versus 87.0%	*P* = 1.0. 26.4 versus 25.2%	*P* = 0.27	Not evaluated^*^	*P* = 0.21. 20.1 versus 14.5%	*P* = 0.11. 9.5 versus 4.6%

Halo absence	*P* = 0.40. 7.8 versus 3.7%	*P* = 0.54. 62.3 versus 66.6%	**X**	<0.0001. 1.8 versus 29.8%	*P* = 0.50	*P* = 0.30. 66.0 versus 58.1%	*P* = 1.0. 18.4 versus 17.0%	*P* = 1.0. 7.5 versus 7.8%

Irregular margins	*P* = 0.51. 9.0 versus 7.0%	*P* = 1.0. 67.0 versus 65.4%	*P* < 0.0001. 99.0 versus 81.8%	**X**	*P* = 0.80	*P* = 0.64. 57.0 versus 60.0%	*P* = 0.77. 22.0 versus 20.4%	*P* < 0.0001. 18.0 versus 4.2%

Elasticity	*P* = 0.89	*P* = 0.27	*P* = 0.50	*P* = 0.80	**X**	*P* = 0.003	*P* = 0.001	*P* = 0.0001

Solid character	*P* = 0.43. 8.3 versus 5.9%	Not evaluated^*^	*P* = 0.30. 84.6 versus 88.6%	*P* = 0.64. 25.0 versus 27.4%	*P* = 0.003	**X**	*P* = 0.11. 15.4 versus 22.3%	*P* = 1.0. 7.9 versus 8.2%

Microcalcifications	*P* = 0.21. 11.4 versus 6.7%	*P* = 0.21. 72.9 versus 64.4%	*P* = 1.0. 87.1 versus 86.0%	*P* = 0.77. 31.4 versus 27.4%	*P* = 0.001	*P* = 0.11. 50.0 versus 61.3%	**X**	*P* < 0.0001. 21.4 versus 4.5%

Macrocalcifications	*P* = 0.01. 21.4 versus 6.7%	*P* = 0.11. 80.0 versus 64.8%	*P* = 1.0. 86.2 versus 86.6%	*P* < 0.0001. 60.0 versus 3.4%	*P* = 0.0001	*P* = 1.0. 60.0 versus 59.2%	*P* < 0.0001. 50.0 versus 4.2%	**X**

^*^All lesions with cystic component were considered as those with heterogeneous echogenicity; thus lesions considered hypoechogenic were all solid ones.

**Table 3:** Sensitivity, specificity, positive predictive value, and odds ratio for all possible panels composed of two markers of malignancy included in our study.

		Cancers possessing both features	Benignancies possessing both features	Sensitivity	Specificity	Positive predictive value	Relative risk
Hypoechogenicity	Taller than wide	6	15	0.27	0.96	0.29	6.75
Hypoechogenicity	Halo absence	20	210	0.91	0.43	0.09	1.61
Hypoechogenicity	Irregular margins	15	63	0.68	0.83	0.19	4.02
Hypoechogenicity	Mean stiffness ≥ 38 kPa	19	67	0.86	0.82	0.22	4.78
Hypoechogenicity	Solid character	18	152	0.82	0.59	0.11	2.00
Hypoechogenicity	Microcalcifications	9	51	0.41	0.86	0.15	2.98
Hypoechogenicity	Macrocalcifications	4	24	0.18	0.94	0.14	2.81
Taller than wide	Halo absence	6	25	0.27	0.93	0.19	4.05
Taller than wide	Irregular margins	5	8	0.23	0.98	0.38	10.54
Taller than wide	Mean stiffness ≥ 38 kPa	6	9	0.27	0.98	0.40	11.24
Taller than wide	Solid character	5	17	0.23	0.95	0.23	4.96
Taller than wide	Microcalcifications	3	8	0.14	0.98	0.27	6.32
Taller than wide	Macrocalcifications	0	6	0.00	0.98	0.00	0.00
Halo absence	Irregular margins	16	95	0.73	0.74	0.14	2.84
Halo absence	Mean stiffness ≥ 38 kPa	19	99	0.86	0.73	0.16	3.24
Halo absence	Solid character	18	181	0.82	0.51	0.09	1.68
Halo absence	Microcalcifications	10	61	0.45	0.84	0.14	2.76
Halo absence	Macrocalcifications	5	26	0.23	0.93	0.16	3.24
Irregular margins	Mean stiffness ≥ 38 kPa	15	35	0.68	0.91	0.30	7.23
Irregular margins	Solid character	15	53	0.68	0.86	0.22	4.77
Irregular margins	Microcalcifications	10	22	0.45	0.94	0.31	7.67
Irregular margins	Macrocalcifications	4	18	0.18	0.95	0.18	3.75
Mean stiffness ≥ 38 kPa	Solid character	17	60	0.77	0.84	0.22	4.78
Mean stiffness ≥ 38 kPa	Microcalcifications	9	33	0.41	0.91	0.21	4.60
Mean stiffness ≥ 38 kPa	Macrocalcifications	4	18	0.18	0.95	0.18	3.75
Solid character	Microcalcifications	9	35	0.41	0.91	0.20	4.34
Solid character	Macrocalcifications	3	18	0.14	0.95	0.14	2.81
Microcalcifications	Macrocalcifications	3	15	0.14	0.96	0.17	3.37

**Table 4:** Relative risks (RRs), sensitivities, and specificities of selected panels composed of three or four markers of malignancy.

Feature 1	Feature 2	Feature 3	Feature 4	Risk ratio	Sensitivity	Specificity
Best panels composed of four features						
Elast	Micro	Ttw	Irreg margins	25.3 (4.5–143.7)	13.6%	99.5%
Elast	Hypo	Ttw	Irreg margins	42.2 (8.7–205.2)	22.7%	99.5%
Elast	Micr	Ttw	Solid	25.3 (4.5–143.7)	13.6%	99.5%
Elast	Ttw	Irreg margins	Solid	28.1 (7.2–110.1)	22.7%	99.2%
Elast	Micr	Irreg margins	Solid	16.9 (7.0–40.7)	36.4%	97.8%
Elast	Hypo	Ttw	Solid	28.1 (7.2–110.1)	22.7%	99.2%

Best panels composed of three features						
Elast	Ttw	Irreg marg	x	16.9 (3.6–78.8)	13.6%	99.2%
Elast	Ttw	Solid	x	10.9 (5.8–20.4)	50.0%	95.4%
Elast	Ttw	Hypo	x	20.2 (6.7–61.2)	27.3%	98.7%
Elast	Irreg	Hypo	x	12.6 (6.9–23.3)	54.5%	95.7%

Selected panels including dependent features						
Elast	Hypo	Micr	Macr	nc	0.0%	97.8%
Elast	Hypo	Macr	Irreg margins	4.2 (0.9–18.7)	9.1%	97.8%
Elast	Micr	Macr	Ttw	nc	0.0%	99.5%
Elast	Micr	Macr	Solid	2.4 (0.3–18.7)	4.5%	98.1%
Elast	Macr	Irreg margins	Solid	4.2 (0.9–18.7)	9.1%	97.8%
Micr	Macr	Ttw	x	nc	0.0%	99.5%
Micr	Macr	Elast	x	1.7 (0.2–12.6)	4.5%	97.3%

Elast: increased stiffness in elastographic examination (mean stiffness ≥ 38 kPa). Hypo: hypoechogenicity. Ttw: “taller-than-wide” feature. Micr: microcalcifications. Macr: macrocalcifications. Irreg margins: irregular margins. Solid: solid character of the lesion; nc: not calculable (none of the cancers possessed all features included in the panel).

## References

[1] Tan G. H., Gharib H. (1997). Thyroid incidentalomas: management approaches to nonpalpable nodules discovered incidentally on thyroid imaging. *Annals of Internal Medicine*.

[2] Staničić J., Prpić M., Jukić T., Borić M., Kusić Z. (2009). Thyroid nodularity—true epidemic or improved diagnostics. *Acta Clinica Croatica*.

[3] Ruchała M., Szczepanek-Parulska E., Fularz M., Woliński K. (2012). Risk of neoplasms in acromegaly. *Wspolczesna Onkologia*.

[4] Wolinski K., Czarnywojtek A., Ruchala M. (2014). Risk of thyroid nodular disease and thyroid cancer in patients with acromegaly—meta-analysis and systematic review. *PLoS ONE*.

[5] Carpi A., Rossi G., Romani R. (2012). Are risk factors common to thyroid cancer and nodule? A forty years observational time-trend study. *PLoS ONE*.

[6] Zou S., Wu F., Guo C. (2012). Iodine nutrition and the prevalence of thyroid disease after salt iodization: a cross-sectional survey in shanghai, a coastal area in China. *PLoS ONE*.

[7] Ruchała M., Szczepanek E. (2010). Thyroid ultrasound—a piece of cake?. *Endokrynologia Polska*.

[8] Woliński K., Szkudlarek M., Szczepanek-Parulska E., Ruchała M. (2014). Usefulness of different sonographic features of malignancy in prediction of the character of thyroid lesions—a meta-analysis of prospective studies involving 5439 nodules. *Polskie Archiwum Medycyny Wewnętrznej*.

[9] Papini E., Pacella C. M., Laszlo L. (2014). Thyroid ultrasound and ultrasound-assisted procedures. From the shadows into an array of applications. *European Journal of Endocrinology*.

[10] Szczepanek-Parulska E., Woliński K., Stangierski A. (2013). Comparison of diagnostic value of conventional ultrasonography and shear wave elastography in the prediction of thyroid lesions malignancy. *PLoS ONE*.

[11] Bojunga J., Dauth N., Berner C. (2012). Acoustic radiation force impulse imaging for differentiation of thyroid nodules. *PLoS ONE*.

[12] Bojunga J., Herrmann E., Meyer G., Weber S., Zeuzem S., Friedrich-Rust M. (2010). Real-time elastography for the differentiation of benign and malignant thyroid nodules: a meta-analysis. *Thyroid*.

[13] Gietka-Czernel M., Kochman M., Bujalska K., Stachlewska-Nasfeter E., Zgliczyński W. (2010). Real-time ultrasound elastography—a new tool for diagnosing thyroid nodules. *Endokrynologia Polska*.

[14] Cantisani V., Ulisse S., Guaitoli E. (2012). Q-elastography in the presurgical diagnosis of thyroid nodules with indeterminate cytology. *PLoS ONE*.

[15] Zhang Y.-F., Xu H.-X., He Y. (2012). Virtual touch tissue quantification of acoustic radiation force impulse: a new ultrasound elastic imaging in the diagnosis of thyroid nodules. *PLoS ONE*.

[16] Trimboli P., Guglielmi R., Monti S. (2012). Ultrasound sensitivity for thyroid malignancy is increased by real-time elastography: a prospective multicenter study. *Journal of Clinical Endocrinology and Metabolism*.

[17] Vorländer C., Wolff J., Saalabian S., Lienenlüke R. H., Wahl R. A. (2010). Real-time ultrasound elastography—a noninvasive diagnostic procedure for evaluating dominant thyroid nodules. *Langenbeck's Archives of Surgery*.

[18] Szczepanek-Parulska E., Woliński K., Stangierski A., Gurgul E., Ruchała M. (2014). Biochemical and ultrasonographic parameters influencing thyroid nodules elasticity. *Endocrine*.

[19] Bhatia K. S. S., Rasalkar D. P., Lee Y. P. (2011). Cystic change in thyroid nodules: a confounding factor for real-time qualitative thyroid ultrasound elastography. *Clinical Radiology*.

